# Elevated troponin levels are associated with early neurological worsening in ischemic stroke with atrial fibrillation

**DOI:** 10.1038/s41598-020-69303-5

**Published:** 2020-07-28

**Authors:** Ki-Woong Nam, Chi Kyung Kim, Sungwook Yu, Jong-Won Chung, Oh Young Bang, Gyeong-Moon Kim, Jin-Man Jung, Tae-Jin Song, Yong-Jae Kim, Bum Joon Kim, Sung Hyuk Heo, Kwang-Yeol Park, Jeong-Min Kim, Jong-Ho Park, Jay Chol Choi, Man-Seok Park, Joon-Tae Kim, Kang-Ho Choi, Yang Ha Hwang, Woo-Keun Seo, Kyungmi Oh

**Affiliations:** 1 Department of Neurology, Seoul National University College of Medicine, Seoul, South Korea; 2 Department of Neurology, Korea University Guro Hospital, Korea University College of Medicine, 148 Gurodong-ro, Guro-gu, Seoul, 08308 South Korea; 3 Department of Neurology, Korea University Anam Hospital, Korea University College of Medicine, Seoul, South Korea; 4 Department of Neurology, Samsung Medical Center, Sungkyunkwan University School of Medicine, 81, Irwon-Ro, Gangnam-Gu, Seoul, 06351 South Korea; 5 Department of Neurology, Korea University Ansan Hospital, Korea University College of Medicine, Ansan, South Korea; 6 Department of Neurology, Ewha Womans University, School of Medicine, Seoul, South Korea; 7 Department of Neurology, The Catholic University of Korea, Seoul, South Korea; 8 Department of Neurology, Kyung Hee University College of Medicine, Seoul, South Korea; 9 Department of Neurology, Chung-Ang University College of Medicine, Chung-Ang University Hospital, Seoul, South Korea; 10 Department of Neurology, Myongji Hospital, Hanyang University College of Medicine, Seoul, South Korea; 11 Department of Neurology, Jeju National University, Jeju, South Korea; 12 Department of Neurology, Chonnam National University Hospital, Chonnam, South Korea; 13 Department of Neurology, Chonnam National University Hwasun Hospital, Hwasun, South Korea; 14 Department of Neurology, Kyungpook National University Hospital, Daegu, South Korea

**Keywords:** Stroke, Atrial fibrillation

## Abstract

Serum cardiac troponin I (cTnI) is often elevated in patients with ischemic stroke, and is associated with their prognosis. Since cTnI is also closely related to atrial fibrillation (AF), cTnI may be a sensitive prognostic indicator in patients with AF-related stroke. This study aimed to evaluate the association between serum cTnI and early neurological deterioration (END) in patients with AF-related stroke. We included consecutive AF-related stroke patients between 2013 and 2015. END was defined as an increase ≥ 2 in the total NIHSS score or ≥ 1 in the motor NIHSS score within the first 72 h of admission. A total of 1,133 patients with AF-related stroke were evaluated. In multivariable analysis, cTnI [adjusted odds ratio (aOR) = 1.16, 95% confidence interval (CI) 1.00–1.34; *P* = 0.047] remained significant after adjusting for confounders. Initial NIHSS score (aOR = 1.03, 95% CI 1.00–1.06; *P* = 0.043) was also positively associated with END; meanwhile, the use of anticoagulants was negatively associated in both vitamin K antagonists (aOR = 0.35, 95% CI 0.23–0.54; *P* < 0.001) and new oral anticoagulants (aOR = 0.41, 95% CI 0.19–0.89; *P* = 0.024). In conclusion, higher serum cTnI was associated with END in patients with AF-related stroke.

## Introduction

Atrial fibrillation (AF) is a well-known risk factor for ischemic stroke^1,2^. AF not only increases the risk of ischemic stroke but is also associated with worse prognosis after events^1–4^. Ischemic stroke patients with coexisting AF (e.g., AF-related stroke) have higher initial NIH Stroke Scale (NIHSS) scores and poor short- and long-term outcomes^2,3,5^. Thus, efforts have been made to identify high-risk groups among AF-related stroke patients, but mostly relying on clinical markers^6^. It would be helpful if we had an objective and reliable biomarker.

Cardiac troponin I (cTnI) is an intracellular protein that controls calcium-mediated myocardial contraction^7,8^. With high sensitivity and specificity, elevated cTnI levels have recently been included in the universal definition of acute myocardial infarction (MI)^9^. Interestingly, elevated cTnI is also commonly found in up to 34% of the acute ischemic stroke patients^10–13^. The exact reasons why cTnI increases during the acute stage of ischemic stroke are still unclear. However, previous studies reported that elevated serum cTnI was associated with initial severe stroke and poor prognosis^7,11,13–18^. In these backgrounds, the American Heart Association (AHA)/American Stroke Association (ASA) guidelines recently recommended the evaluation of cTnI levels in all patients with acute ischemic stroke in consideration of the pathological overlap between cardiovascular and cerebrovascular diseases and their clinical impact on the prognosis of ischemic stroke^19^.

Patients with AF have vulnerable cardiac environments with increased oxygen demand due to tachycardia and decreased myocardial oxygen supply due to the shortening of diastole^8,13^. In these patients, cTnI reflects AF-related cardiac structural changes [e.g., left atrial (LA) enlargement, endothelial dysfunction, and fibrosis] and secondary thrombus formation (e.g., LA thrombus, and spontaneous echocardiographic contrast)^20,21^. Furthermore, elevated cTnI is associated with poor prognosis in patients with AF^20,22,23^. Thus, serum cTnI may be closely related to the prognosis of AF-related stroke patients with both AF and ischemic stroke. In this study, we aimed to evaluate the association between serum cTnI and early neurological deterioration (END) in patients with AF-related stroke.

## Results

A total of 1,133 patients with AF-related stroke were evaluated. The mean age of the cohort was 74 years, and 51.1% were male. The median initial NIHSS score was 10 [3–16]. END occurred in 164 (14.5%) patients and the median time to admission was 0 [0–1] day. The median cTnI level was 0.02 [0.02–0.04] ng/mL. Other baseline characteristics are presented in Supplementary Table 1.

In univariate analysis, END was significantly associated with older age, higher frequencies of hypertension and previous stroke history, lesser use of anticoagulants, and higher initial NIHSS score and serum cTnI levels (Table 1). In multivariable logistic regression analysis, cTnI [adjusted odds ratio (aOR) = 1.16, 95% confidence interval (CI) 1.00–1.34, *P* = 0.047] remained significant after adjusting for confounders. Furthermore, initial NIHSS score (aOR = 1.03, 95% CI 1.00–1.06; *P* = 0.043) was positively associated with END, independent of cTnI (Table 2). The use of anticoagulants was negatively associated with END in both vitamin K antagonists (VKA) (aOR = 0.35, 95% CI 0.23–0.54; *P* < 0.001) and new oral anticoagulants (NOAC) (aOR = 0.41, 95% CI 0.19–0.89; *P* = 0.024) (Table 3). These positive and negative associations continued when the variable “cTnI > 0.03 ng/mL” was used to conduct additional sensitivity analyses (Table 3).

**Table 1:** Baseline characteristics of the with and without END groups.

	Non-END (n = 969)	END (n = 164)	*P*-value
Age, years [IQR]	74 [68–80]	76 [71–81]	0.018
Sex, male (%)	510 (52.6)	69 (42.1)	0.012
Body mass index, kg/m^2^ [SD]	23.1 ± 3.3	23.0 ± 3.3	0.687
Hypertension, n (%)	643 (66.4)	124 (75.6)	0.019
Diabetes, n (%)	236 (24.4)	41 (25.0)	0.859
Hyperlipidemia, n (%)	131 (13.5)	28 (17.1)	0.226
Stroke history, n (%)	243 (25.1)	56 (34.1)	0.015
**Atrial fibrillation type, n (%)**			0.087
Paroxysmal	236 (24.4)	50 (30.7)	
Sustained	732 (75.6)	113 (69.3)	
Initial NIHSS score [IQR]	10 [3–16]	13 [7–19]	< 0.001
Thrombolysis, n (%)	334 (34.5)	54 (32.9)	0.700
**Use of anticoagulants, n (%)**			< 0.001
No use	211 (21.8)	75 (45.7)	< 0.001
Vitamin K antagonist	679 (70.1)	79 (48.2)	< 0.001
New oral anticoagulant	79 (8.2)	10 (6.1)	0.366
**Systolic BP, mmHg [IQR]**	140 [120–160]	140 [130–160]	0.068
**Diastolic BP, mmHg [IQR]**	85 [74–97]	82 [80–98]	0.418
**Fasting blood sugar, mg/dL [IQR]**	114 [98–137]	120 [102–146]	0.051
**HbA1c, % [IQR]**	5.7 [5.3–6.2]	5.8 [5.4–6.5]	0.083
**Total cholesterol, mg/dL [SD]**	162 ± 40	167 ± 39	0.202
**White blood cells, × 10**^**3**^**/μL [IQR]**	7.83 [6.20–9.80]	7.89 [6.43–10.50]	0.153
**CK-MB, ng/mL [IQR]**	2.13 [1.23–3.60]	2.11 [1.23–3.86]	0.840
**Troponin I, ng/mL [IQR]**	0.02 [0.02–0.03]	0.02 [0.02–0.06]	0.005

*END *early neurological deterioration, *NIHSS *National Institutes of Health Stroke Scale, *BP *blood pressure, *CK-MB *creatinine kinase MB fraction.

**Table 2:** Multivariable logistic regression analysis of possible predictors of early neurological deterioration.

	Crude OR (95% CI)	*P-*value	Adjusted OR (95% CI)	*P-*value
Age	1.02 [1.00–1.04]	0.061	1.00 [0.98–1.02]	0.987
Sex	0.65 [0.47–0.91]	0.013	0.69 [0.46–1.03]	0.069
Hypertension	1.57 [1.07–2.30]	0.020	1.37 [0.88–2.13]	0.171
Stroke history	1.55 [1.09–2.21]	0.015	1.51 [1.00–2.28]	0.050
Sustained atrial fibrillation	1.37 [0.95–1.98]	0.088	1.18 [0.72–1.93]	0.522
Initial NIHSS score	1.05 [1.03–1.08]	< 0.001	1.03 [1.00–1.06]	0.043
**Use of anticoagulants**		< 0.001		< 0.001
No use	Ref	Ref	Ref	Ref
Vitamin K antagonist	0.33 [0.23–0.47]	< 0.001	0.35 [0.23–0.54]	< 0.001
New oral anticoagulant	0.36 [0.18–0.72]	0.004	0.41 [0.19–0.89]	0.024
**Systolic blood pressure**	1.01 [1.00–1.01]	0.032	1.00 [1.00–1.01]	0.409
Fasting blood sugar*	1.41 [0.77–2.60]	0.266	0.89 [0.48–1.65]	0.705
Troponin I*	1.22 [1.07–1.38]	0.002	1.16 [1.00–1.34]	0.047

*NIHSS *National Institutes of Health Stroke Scale.

*This variable was transformed into a log scale.

**Table 3:** Multivariable logistic regression analysis of possible predictors of early neurological deterioration using cTnI > 0.03 ng/mL.

	Crude OR (95% CI)	*P-*value	Adjusted OR (95% CI)	*P-*value
Age	1.02 [1.00–1.04]	0.061	1.00 [0.98–1.02]	0.975
Sex	0.65 [0.47–0.91]	0.013	0.69 [0.46–1.03]	0.069
Hypertension	1.57 [1.07–2.30]	0.020	1.35 [0.86–2.10]	0.191
Stroke history	1.55 [1.09–2.21]	0.015	1.50 [0.99–2.27]	0.055
Sustained atrial fibrillation	1.37 [0.95–1.98]	0.088	1.20 [0.73–1.96]	0.484
Initial NIHSS score	1.05 [1.03–1.08]	< 0.001	1.03 [1.00–1.06]	0.054
**Use of anticoagulants**		< 0.001		< 0.001
No use	Ref	Ref	Ref	Ref
Vitamin K antagonist	0.33 [0.23–0.47]	< 0.001	0.35 [0.23–0.53]	< 0.001
New oral anticoagulant	0.36 [0.18–0.72]	0.004	0.40 [0.19–0.87]	0.020
**Systolic blood pressure**	1.01 [1.00–1.01]	0.032	1.00 [1.00–1.01]	0.511
Fasting blood sugar*	1.41 [0.77–2.60]	0.266	0.87 [0.47–1.61]	0.651
**Troponin I > 0.03**	1.74 [1.23–2.45]	0.002	1.59 [1.07–2.37]	0.023

*NIHSS *National Institutes of Health Stroke Scale.

*This variable was transformed into a log scale.

When the relationship between serum cTnI and vascular risk factors/echocardiographic parameters was analyzed, cTnI showed a positive association with the initial NIHSS scores, regional wall motion abnormalities, and LA thrombus, and negative associations with body mass index and left ventricular ejection fraction (LVEF) (Table 4). In addition, the END group showed more frequent 7d-poor outcome (80.0% versus 44.9%, *P* < 0.001) and 3m-poor outcome (78.3% versus 41.6%, *P* < 0.001), showing an effect on subsequent prognosis (Table 5).

**Table 4:** Univariate linear regression analysis between cardiac troponin I* and vascular risk factors/echocardiographic parameters.

	β (95% CI)	*P-*value
Age	0.004 (− 0.003 to 0.012)	0.216
Sex	− 0.106 (− 0.240 to 0.028)	0.120
Body mass index	− 0.034 (− 0.055 to − 0.013)	0.002
Hypertension	0.022 (− 0.121 to 0.165)	0.764
Diabetes	0.042 (− 0.114 to 0.198)	0.595
Hyperlipidemia	− 0.029 (− 0.222 to 0.164)	0.766
Stroke history	0.098 (− 0.054 to 0.249)	0.207
Sustained atrial fibrillation	− 0.061 (− 0.215 to 0.094)	0.441
Initial NIHSS score	0.022 (0.013 to 0.030)	< 0.001
LVEF	− 0.017 (− 0.024 to − 0.010)	< 0.001
Deceleration time*	− 0.006 (− 0.306 to 0.294)	0.968
E/e′ ratio*	0.138 (− 0.121 to 0.397)	0.296
**Wall motion abnormality**		
No abnormality	Ref	Ref
Regional	0.608 (0.206 to 1.011)	0.003
Global	0.225 (− 0.135 to 0.585)	0.220
**Left atrial thrombus**	0.599 (0.111 to 1.088)	0.016

*NIHSS *National Institutes of Health Stroke Scale, *LVEF *left ventricular ejection fraction.

*This variable was transformed into a log scale.

**Table 5:** Prognosis comparison between patients with and without END.

	Non-END (n = 969)	END (n = 164)	*P*-value
7d-mRS score [IQR]	3 [1–5]	5 [4–6]	< 0.001
7d-poor outcome, n (%)*	425 (44.9)	124 (80.0)	< 0.001
3m-mRS score [IQR]	3 [1–5]	5 [4–6]	< 0.001
3m-poor outcome, n (%)*	342 (41.6)	101 (78.3)	< 0.001

*mRS *modified Rankin Scale.

*7d- and 3m-Poor outcome was defined based on mRS score > 3.

## Discussion

In this study, we found that high serum cTnI levels were associated with END in patients with AF-related stroke. Furthermore, cTnI was not associated with any avascular risk factors, but was associated with LVEF or regional wall motion abnormality (Table 4). Thus, the close relationship between the two seems to be linked by more cardio-specific mechanisms.

The exact mechanisms explaining the close relationship between cTnI and END are unclear. Serum cTnI is a simple indicator of damaged myocytes, and it is difficult to say that it is a substance that can cause END in itself. Therefore, it is reasonable to say that the heart was damaged by any causes, and that the decreased cardiac function caused END. We found the cause of the cardiac dysfunction in (1) neurogenic-heart syndrome after stroke and (2) hidden cardiac disease before stroke.

First, we can think about how cTnI is associated with END in neurogenic-heart syndrome condition after a stroke. In this situation, increased cTnI may reflect a severe stroke that is vulnerable to early progression. When an ischemic stroke occurs, circulating catecholamine increases with activation of the hypothalamus-pituitary gland-adrenal gland axis^7,10,24,25^. This increased catecholamine injures myocytes via calcium channel activation, called “myocytolysis”, and elevates serum cTnI levels^7,10,12,24–26^. At this time, the degree of cTnI level increase is proportional to the severity of index stroke, which can be confirmed in our data (Table 4)^11,17,22,25,26^. The severity of index stroke, which is represented by initial NIHSS score, is the most well-known risk factor of END^27^. Eventually, it can be interpreted that elevated cTnI serves as a marker for patients with severe stroke who are originally well progressed. However, the authors also thought about the possibility of elevated cTnI linking to END through a mechanism other than simply reflecting severe stroke. To prove this, an additional subgroup analysis was performed on the patients with mild (initial NIHSS score < 5) AF-related stroke. Even in the situation where the effect of the initial stroke severity was limited to a minimum, cTnI was closely related to END (Supplementary Table 2). Therefore, the relationship between two seems to be also explained by a mechanism other than the initial stroke severity. On the one hand, catecholamine-induced myocardial damage can also provoke transient left ventricular dysfunction, called Takotsubo cardiomyopathy^20,24,28–30^. In previous studies, ischemic stroke patients with Takotsubo cardiomyopathy had worse initial clinical outcomes, including END. Also, like those with Takotsubo cardiomyopathy, our data showed that patients with elevated cTnI were closely related to systolic dysfunction (e.g., LVEF) or regional wall motion abnormality and not to diastolic dysfunction (e.g., DT, E/e′) or global wall motion abnormality (Table 4)^28–30^. Thus, it may not be involved in all patients with END, but we suggest that Takotsubo cardiomyopathy may be a sufficient mechanism to explain this phenomenon.

Next, the possibility of hidden cardiac disease before stroke can be considered. Patients with ischemic stroke are often unaware of accompanying heart disease due to symptom masking by the stroke itself^7,11,24,31^. These hidden cardiovascular diseases could lead to END, at the same time increasing serum cTnI levels. Additionally, as seen in the Troponin Elevation in Acute Ischemic Stroke (TRELAS) study, unknown coronary culprit lesions are found in up to 25% of the ischemic stroke patients^32^. These lesions are chronic but unstable, able to cause problems at any time^32^. It is difficult to know whether these heart diseases were exactly accompanied by the data we present in our cohort. However, given the high prevalence of accompanying heart disease in previous studies, this is quite possible.

Interestingly, in this study, the use of early anticoagulant showed a negative correlation with the occurrence of END. This negative correlation was found in both VKA and NOAC regardless of the type of anticoagulant. Simply, it can be thought that the use of anticoagulant suppressed further embolism in the acute period, which resulted in less END. However, on the contrary, it may be a result of the tendency not to use anticoagulants in severe strokes that are good at early progression. Actually, our data showed that the patients without anticoagulant showed higher initial NIHSS score and more frequent hemorrhagic transformation than the rest of the patients (Supplementary Table 3). This study is a cross-sectional study, so it is difficult to obtain further information on causal relationships or related information. Further prospective studies addressing the relationship between the use or type of early anticoagulant and the development of END would provide an impression that is clinically interesting.

The sensitivity, specificity, positive predictive value, and negative predictive value based on cTnI > 0.03 ng/mL, which we used as the reference value of sensitivity analysis, can be seen relatively high specificity and negative predictive values (Supplementary Fig. 2). This seems to mean that the clinical probability of END is very low if serum cTnI is lower than 0.03 ng/mL. However, relatively low sensitivity and positive predictive value also mean that cut-off point 0.03 ng/mL is somewhat high to be used as a screening tool for END. It may be necessary to consider the new appropriate reference value.

Our study had several limitations. First, this study was designed as a retrospective study. Although we included large numbers of participants at multiple centers, selection bias may have occurred. Second, due to the limitations of cross-sectional analysis, we could only indicate association, not causality. Further large, prospective studies are needed to confirm the causality. Third, we defined END by a relatively sensitive definition^33^. Thus, the END events could have been overestimated. However, this sensitive definition of END has been validated in previous studies^34^, and it also has a significant effect on further prognosis (e.g., 7d- and 3m-poor outcome) in our data. Therefore, it is believed that our END definition will have no problems using it. Fourth, this study did not include information related to imaging parameters. If there was information on lesions and vascular occlusion on the initial MRI and follow-up MRI images within 72 h, it would be helpful to infer the mechanism of END occurrence. Fifth, we analyzed the relationship with END using only baseline cTnI values at the time of admission. If information such as the change in cTnI within 72 h and its highest value can be known, it is likely to be able to analyze the more certain relationship between cTnI and END and infer its mechanism. Lastly, considering the definition of END, we included ischemic stroke patients within 72 h from symptom onset. Therefore, if the END event occurs before the patient visits, it may be underestimated than the actual prevalence of END. However, since most of the participants (91.5%) in our dataset visited within 24 h, there will be very few missing cases that actually happen, and not enough to affect our main results.

We demonstrated that high serum cTnI levels at admission were associated with END events in patients with AF-related stroke using data from 11 large centers in Korea. These positive associations were observed within the range of cTnI levels recognized as “subclinical”. Thus, even slight increases in cTnI should be cautiously interpreted and the patients need close observation. However, our insights must be validated with further large, prospective studies.

## Methods

### Patients and population

The current study is a sub-study of K-ATTENTION (Korean ATrial fibrillaTion EvaluatioN regisTry in Ischemic strOke patieNts) that was a real-world cohort composed of prospective stroke registries from 11 large centers in South Korea between January 2013 and December 2015 (n = 3,213)^35^. The K-ATTENTION study aimed to investigate the relationships between the use of oral anticoagulants and outcomes of AF-related stroke patients^35^. From this large dataset, we included consecutive AF-related stroke patients who were admitted within 72 h after symptom onset (n = 2,960). The exclusion criteria were patients without (1) END data (n = 771) or (2) serum cTnI levels (n = 821) or (3) patients who suffered severe heart diseases (e.g., congestive heart failure, MI, severe valvular heart disease, etc.) (n = 235). A total of 1,133 patients were included in the final analyses (Supplementary Fig. 1). All patients were evaluated and treated according to the individual center’s protocol, including brain magnetic resonance imaging, echocardiography, and laboratory evaluations.

This retrospective study was approved by the Institutional Review Board (IRB) at Samsung Medical Center (SMC-2016-07-011). The requirement to obtain written informed consent from the participants was exempted by the IRB due to the retrospective design using anonymous and de-identified information. All experiments were performed in accordance with the Declaration of Helsinki and relevant guidelines and regulations.

### Clinical assessment

We evaluated demographic factors, clinical factors, and vascular risk factors, including age, sex, body mass index, hypertension, diabetes, hyperlipidemia, previous stroke history, initial NIHSS score, types of AF, thrombolysis therapy, the use of anticoagulants, and systolic/diastolic blood pressure^34^. The initial NIHSS score was rated daily from admission to discharge by well-trained neurologists who were not involved in the study^34^ AF was documented by electrocardiogram, 24-h Holter monitoring, and/or continuous electrocardiogram monitoring during hospitalization. The types of AF were classified as paroxysmal or sustained. The use of anticoagulants was defined as using any type of these drugs during the acute period within 72 h from admission. Considering the difference in acute period pharmacological action according to the type of anticoagulant, we divided the entire participants into 3 groups: no use, VKA, and NOAC. Patients who used intravenous anticoagulants were excluded from the initial process because K-ATTENTION study, which corresponds to the original study of this study, was intended to examine the clinical prognosis of oral anticoagulants in AF-related stroke patients^35^. Laboratory examinations, including glucose profiles, lipid profiles, complete blood cell counts, creatinine kinase-MB, and cTnI, were obtained within the first 24 h of admission. As an outcome, END was defined as an increase ≥ 2 in the total NIHSS score or ≥ 1 in the motor NIHSS score within the first 72 h of admission^34^. We also measured the participants’ 7d-mRS and 3m-mRS scores. Based on this score, mRS score > 3 was defined as “poor outcome”^36^.

### Echocardiographic evaluation

Comprehensive 2-dimensional and Doppler echocardiography (TTE) examinations were performed by skilled cardiac sonographers and the findings were interpreted by cardiologists at each center. Considering the differences between the TTE protocols among the centers, we extracted common echocardiographic parameters, including cardiac structural parameters [e.g., LA diameter, left ventricular (LV) end-systolic diameter, LV end-diastolic diameter, interventricular septal dimension, and LV posterior wall thickness], LVEF, deceleration time (DT), the peak trans-mitral filling velocity (E)/mean mitral annular velocity at early diastole (e′) ratio, and the presence of LA thrombus^37^. LVEF was obtained using Simpson’s method with estimations of the end-systolic and end-diastolic LV volume^37^. E and DT were measured by the pulse wave Doppler method at the tip of the mitral leaflets from an apical 4-chamber view^37,38^. Tissue Doppler imaging was applied to the apical 4-chamber view at the septal mitral annulus to determine e′. To calculate the E/e′ ratio, the maximum velocity of the E-wave of mitral valve inflow was divided by the maximum velocity of e′^37,38^.

### Statistical analysis

All statistical analyses were performed using SPSS version 20.0 (IBM, SPSS, Chicago, IL, USA). Univariate analyses for assessing the possible predictors of END were performed using Student’s *t*-test or the Mann–Whitney *U*-test for continuous variables. Variables with severely skewed data were transformed into a log scale. The Chi-squared test or Fisher’s exact test was used for categorical variables. Based on the results of the univariate analyses, variables with *P* < 0.10 were introduced into the multivariable logistic regression analysis. To confirm our results, we conducted additional sensitivity analysis using the variable of “cTnI > 0.03 ng/mL” that has been used in previous studies^10,13,18^.

To understand the underlying pathological mechanisms between cTnI and END, the relationship between serum cTnI and vascular risk factors/echocardiographic parameters was evaluated using simple linear regression analysis. In addition, in order to examine the effect of END on the subsequent clinical prognosis, we compared the frequencies of 7d- and 3m-poor outcome between END group and non-END group. All variables with *P* < 0.05 were considered significant in this study.

## Supplementary information

Supplementary Information.
