# Supplementary material for: Elevated troponin levels are associated with early neurological worsening in ischemic stroke with atrial fibrillation
Source: Sci Rep. 2020 Jul 28;10:12626. doi: 10.1038/s41598-020-69303-5 (PMC7387448; doi:10.1038/s41598-020-69303-5)
Supplement: Supplementary file 1 — Supplementary Information. [file 41598_2020_69303_MOESM1_ESM.docx]

**Supplemental information**

**Elevated Troponin Levels are Associated With Early Neurological Worsening in Ischemic Stroke with Atrial Fibrillation**

Ki-Woong Nam, MD, MSc^1,*^; Chi Kyung Kim MD, PhD^2,*^; Sungwook Yu, MD, PhD^3^; Jong-Won Chung MD, PhD^4^; Oh Young Bang MD, PhD^4^; Gyeong-Moon Kim, MD, PhD^4^; Jin-Man Jung, MD, PhD^5^; Tae-Jin Song, MD, PhD^6^; Yong-Jae Kim, MD, PhD^7^; Bum Joon Kim, MD, PhD^8^; Sung Hyuk Heo MD, PhD^8^; Kwang-Yeol Park MD, PhD^9^; Jeong-Min Kim, MD, PhD^9^; Jong-Ho Park MD, PhD^10^; Jay Chol Choi, MD, PhD^11^; Man-Seok Park MD, PhD^12^; Joon-Tae Kim MD, PhD^12^; Kang-Ho Choi MD, PhD^13^; Yang Ha Hwang MD, PhD^14^; Woo-Keun Seo, MD, PhD^4^; Kyungmi Oh, MD, PhD^3^

**Supplementary Figure 1. Patient inclusion flow chart**

**
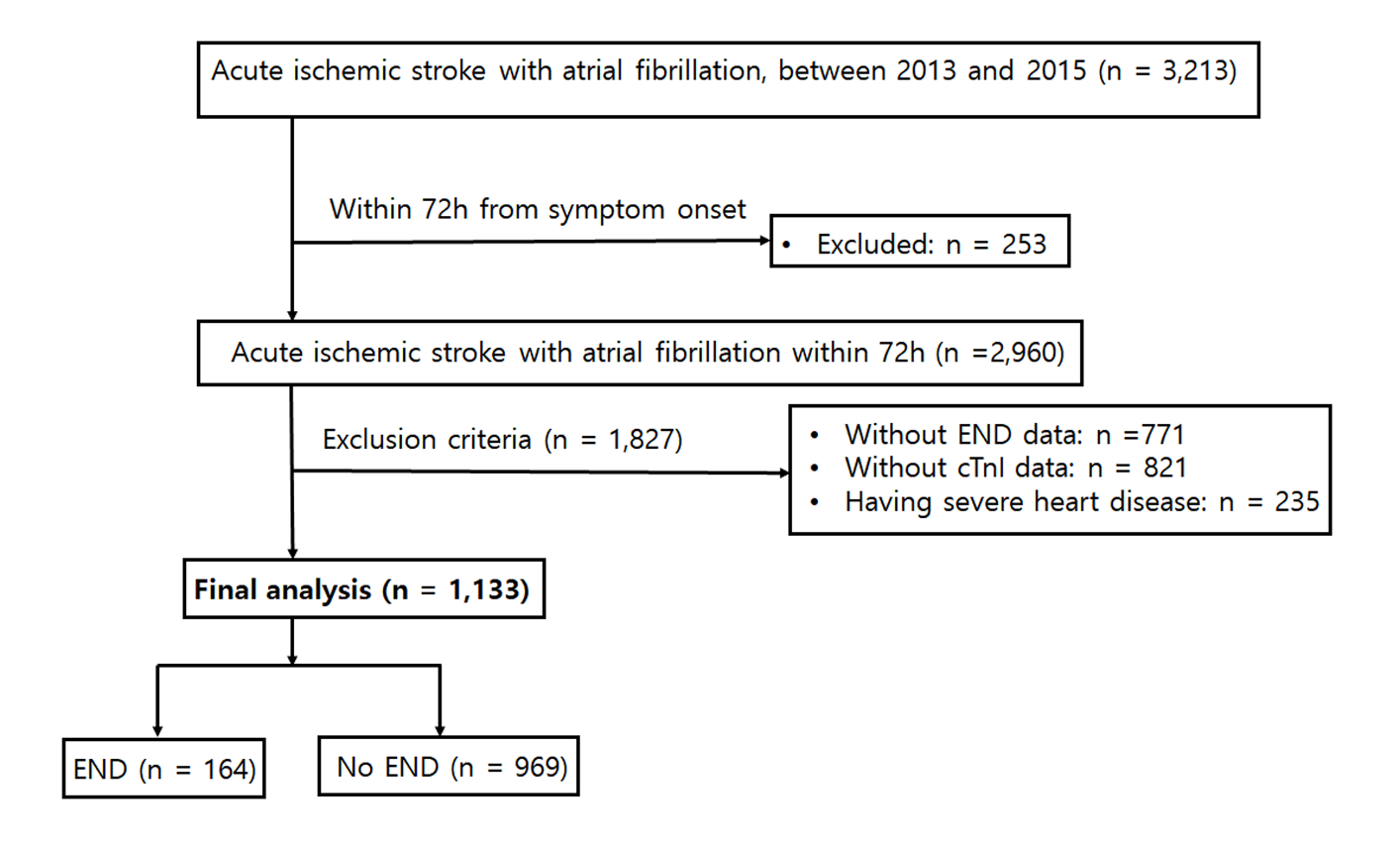
**

**Supplementary Figure 2. Sensitivity, specificity, positive predictive value, and negative predictive value calculated according to the criteria of Troponin I > 0.03 ng/mL**


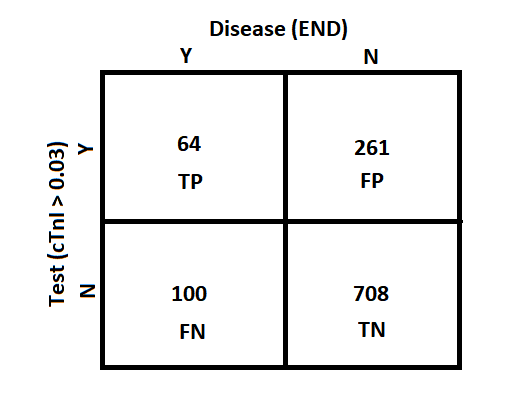


The above figures show that sensitivity is 0.390, specificity is 0.731, positive predictive value is 0.197, and negative predictive value is 0.876.

**Supplementary Table 1. Baseline characteristics of the cohort (total *n* = 1,133)**

| **Clinical findings** |  |
| --- | --- |
| Age, y [SD] | 74 ± 9 |
| Sex, male, n (%) | 579 (51.1) |
| Body mass index, kg/m^2^ [SD] | 23.1 ± 3.3 |
| Hypertension, n (%) | 767 (67.7) |
| Diabetes, n (%) | 277 (24.4) |
| Hyperlipidemia, n (%) | 159 (14.0) |
| Stroke history, n (%) | 299 (26.4) |
| Atrial fibrillation type, n (%) |  |
| Paroxysmal | 845 (74.6) |
| Sustained | 286 (25.2) |
| Initial NIHSS score, [IQR] | 10 [3-16] |
| Thrombolysis, n (%) | 388 (34.2) |
| Use of anticoagulants, n (%) |  |
| No use | 286 (25.2) |
| Vitamin K antagonist | 758 (66.9) |
| New oral anticoagulant | 89 (7.9) |
| Systolic BP, mmHg [SD] | 142 ± 27 |
| Diastolic BP, mmHg [IQR] | 84 [75-97] |
| Early neurological deterioration, n (%) | 164 (14.5) |
| 7d-modified Rankin Scale score | 3 [2-5] |
| 3m-modified Rankin Scale score | 3 [1-5] |
| **Laboratory findings** |  |
| Fasting blood sugar, mg/dL [IQR] | 115 [99-138] |
| HbA1c, % [IQR] | 5.7 [5.4-6.2] |
| Total cholesterol, mg/dL [SD] | 163 ± 40 |
| White blood cells, x10^3^/μL [IQR] | 7.86 [6.22-9.89] |
| CK-MB, ng/mL [IQR] | 2.12 [1.23-3.70] |
| Troponin I, ng/mL [IQR] | 0.02 [0.02-0.04] |
| **Echocardiographic findings** |  |
| Left atrial diameter, mm [SD] | 49.1 ± 8.4 |
| LV end-systolic diameter, mm [IQR] ^*^ | 33.0 [28.3-37.0] |
| LV end-diastolic diameter, mm [IQR] ^*^ | 49.0 [44.2-53.1] |
| Interventricular septal dimension, mm [IQR] ^*^ | 10.0 [9.0-11.0] |
| LV posterior wall thickness, mm [IQR] ^*^ | 10.0 [9.0-10.5] |
| LV ejection fraction, % [IQR] | 60.44 [54.00-65.00] |
| E/e′ ratio [IQR] ^*^ | 12.30 [9.90-16.13] |
| Deceleration time, ms [IQR] ^*^ | 168.25 [141.00-212.74] |
| Wall motion abnormality, n (%) ^*^ |  |
| No abnormality | 326 (28.8) |
| Regional | 35 (3.1) |
| Global | 45 (4.0) |
| Left atrial thrombus, n (%) | 22 (1.9) |
| **Radiological findings** |  |
| Hemorrhagic transformation, n (%) | 216 (19.1) |

NIHSS = National Institutes of Stroke Scale, BP = blood pressure, CK-MB = creatine kinase MB fraction, LV = left ventricle

^*^These variables were measured in 406 participants

**Supplementary Table 2. Multivariable logistic regression analysis of possible predictors of early neurological deterioration in patients with mild initial symptoms (initial NIHSS <5)**

|  | **Crude OR**  **(95% CI)** | ***P-*value** | **Adjusted OR**  **(95% CI)** | ***P-*value** |
| --- | --- | --- | --- | --- |
| Age | 1.03 [0.99-1.08] | 0.165 | 1.05 [0.99-1.12] | 0.103 |
| Sex | 0.92 [0.41-2.08] | 0.847 | 0.90 [0.33-2.42] | 0.828 |
| Hypertension | 1.90 [0.74-4.86] | 0.182 | 1.64 [0.54-4.96] | 0.380 |
| Stroke history | 3.14 [1.39-7.10] | 0.006 | 4.66 [1.74-12.51] | 0.002 |
| Sustained atrial fibrillation | 0.96 [0.35-2.65] | 0.937 | 0.16 [0.02-1.49] | 0.108 |
| Initial NIHSS score | 1.17 [0.86-1.59] | 0.314 | 1.20 [0.83-1.75] | 0.328 |
| Use of anticoagulants |  | 0.390 |  | 0.496 |
| No use | Ref | Ref | Ref | Ref |
| Vitamin K antagonist | 0.51 [0.19-1.37] | 0.184 | 0.99 [0.28-3.46] | 0.990 |
| New oral anticoagulant | 0.47 [0.09-2.47] | 0.370 | 0.26 [0.02-2.91] | 0.275 |
| Systolic blood pressure | 1.00 [0.98-1.01] | 0.822 | 1.00 [0.99-1.02] | 0.669 |
| Fasting blood sugar^*^ | 0.24 [0.03-1.76] | 0.159 | 0.12 [0.01-1.00] | 0.050 |
| Troponin I^*^ | 1.40 [1.02-1.94] | 0.040 | 1.76 [1.19-2.61] | 0.005 |

NIHSS = National Institutes of Health Stroke Scale

^*^This variable was transformed into a log scale

**Supplementary Table 3. Comparisons between groups using and without anticoagulants**

|  | Anticoagulants (-)  (n = 286) | Anticoagulants (+)  (n = 847) | P-value |
| --- | --- | --- | --- |
| Age, years [IQR] | 76 [69-82] | 74 [67-80] | 0.001 |
| Sex, male (%) | 144 (50.3) | 435 (51.4) | 0.768 |
| Body mass index, kg/m2 [SD] | 22.9 [20.7-25.2] | 22.9 [20.8-25.0] | 0.883 |
| Hypertension, n (%) | 200 (69.9) | 567 (66.9) | 0.350 |
| Diabetes, n (%) | 69 (24.1) | 208 (24.6) | 0.883 |
| Hyperlipidemia, n (%) | 32 (11.2) | 127 (15.0) | 0.109 |
| Stroke history, n (%) | 75 (26.2) | 224 (26.4) | 0.941 |
| Atrial fibrillation type, n (%) |  |  | < 0.001 |
| Paroxysmal | 236 (82.8) | 609 (72.0) |  |
| Sustained | 49 (17.2) | 237 (28.0) |  |
| Initial NIHSS score [IQR] | 14 [8-18] | 9 [2-15] | < 0.001 |
| END, n (%) | 75 (26.2) | 89 (10.5) | < 0.001 |
| 7d-Poor outcome, n (%)^*^ | 218 (77.3) | 331 (40.4) | < 0.001 |
| 3m-Poor outcome, n (%)^*^ | 184 (73.3) | 259 (36.9) | < 0.001 |
| CK-MB, ng/mL [IQR] | 2.33 [1.58-4.12] | 2.00 [1.19-3.48] | 0.002 |
| Troponin I, ng/mL [IQR] | 0.02 [0.02-0.04] | 0.02 [0.02-0.04] | 0.027 |
| Hemorrhagic transformation, n (%) | 101 (39.5) | 115 (15.3) | < 0.001 |

NIHSS = National Institutes of Health Stroke Scale, END = early neurological deterioration

^*^7d- and 3m-Poor outcome was defined based on mRS score > 3.
